# The use of the online Inverted Classroom Model for digital teaching with gamification in medical studies

**DOI:** 10.3205/zma001399

**Published:** 2021-01-28

**Authors:** Johanna Huber, Matthias Witti, Michaela Schunk, Martin R. Fischer, Daniel Tolks

**Affiliations:** 1 LMU Klinikum München, Institut für Didaktik und Ausbildungsforschung in der Medizin, Munich, Germany; 2 LMU Klinikum München, Klinik und Poliklinik für Palliativmedizin, Munich, Germany; 3 Leuphana Universität Lüneburg, Zentrum für Gesundheitswissenschaften, Lüneburg, Germany

**Keywords:** online Inverted Classroom Model, ICAP model, game-based learning, medical education, German health system

## Abstract

**Introduction:** In 2014, a newly designed, case-based seminar was successfully implemented in the subjects of health systems, health economics and public health care (GGG). The seminar “The Lonely Patient” is based on a real patient case and deals with the German health care system from the perspective of a patient. In order to create more space for discussion and exchange among students, the seminar was redesigned on the basis of the Inverted Classroom Method (ICM).

**Project description: **Due to the COVID-19 pandemic, new, purely digital teaching formats had to be developed quickly in the sense of Emergency Remote Teaching. Therefore, the Inverted Classroom concept of the seminar was transformed into an online ICM. In order to promote active learning based on the ICAP model (Interactive, Constructive, Active, Passive), the online face-to-face part was designed as a synchronous interactive learner-centered course using the gamified audience response system Kahoot!

**Results: **Evaluation results to date and feedback rounds with students indicate that the online ICM-version of the seminar leads to at least as good evaluation results as the previous face-to-face course. In particular, the students positively emphasize the use of Kahoot! as an activating digital medium.

**Discussion: **Through the use of the ICM and the gamified audience response system Kahoot!, students could be activated in meaningful ways. The resulting discussions about the patient case and teaching content of the quiz questions in the synchronous online course could be implemented just as well as in the classroom-based course of previous semesters.

**Conclusion: **The application of the online ICM, along with the consideration of the ICAP Model, has led to the successful implementation of a digital course within the context of the increased difficulty surrounding the emergency remote teaching. Additionally, students’ learning success has remained at a similar level as during traditional classroom-based courses of previous semesters.

## Zusammenfassung

**Einleitung: **2014 wurde in den Fächern Gesundheitssysteme, -ökonomie und öffentliche Gesundheitspflege (GGG) ein neu konzipiertes, fallbasiertes Seminar erfolgreich implementiert. Das Seminar „Der einsame Patient“ basiert auf einem realen Patientenfall und thematisiert das deutsche Gesundheitssystem aus der Perspektive eines Patienten. Um mehr Raum für Diskussionen und den Austausch der Studierenden untereinander zu gewinnen, wurde das Seminar auf der Basis der Inverted-Classroom-Methode (ICM) umgestaltet.

**Projektbeschreibung:** Durch die COVID-19-Pandemie mussten zügig im Sinne des Emergency Remote Teaching neue, rein digitale Lehrformate entwickelt werden. Daher wurde das Inverted-Classroom-Konzept des Seminars in eine online ICM umgestaltet. Um auf der Basis des ICAP-Modells (Interactive, Constructive, Active, Passive) aktives Lernen zu fördern, wurde die online Präsenzphase mit Hilfe des gamifizierten Audience-Response-Systems Kahoot! als synchrone interaktive Lerner-zentrierte Lehrveranstaltung gestaltet.

**Ergebnisse: **Die bisherigen Evaluationsergebnisse und die Feedbackrunden mit den Studierenden deuten darauf hin, dass die online ICM des Seminars zu mindestens genauso guten Evaluationsergebnissen wie die vorherige Präsenzveranstaltung führt. So heben die Studierenden insbesondere die Nutzung von Kahoot! als aktivierendes digitales Medium positiv hervor.

**Diskussion: **Durch den Einsatz des ICM und des gamifizierten Audience-Response-Systems Kahoot! konnten die Lernenden sinnvoll aktiviert werden. Die dadurch induzierten Diskussionen über den Patientenfall und die Lehrinhalte der Quiz-Fragen im Rahmen der synchronen Online-Lehrveranstaltung konnten genauso gut umgesetzt werden wie in der Präsenzveranstaltung der vorangegangenen Semester.

**Schlussfolgerung: **Die Anwendung des online ICM bei gleichzeitiger Beachtung des ICAP-Modells, hat im Kontext der erschwerten Bedingungen des Emergency Remote Teaching zu einer erfolgreichen Implementierung einer digitalen Lehrveranstaltung geführt. Zudem ist der Lernerfolg der Studierenden auf einem ähnlichen Niveau wie in der Präsenzveranstaltung der vorangegangenen Semester geblieben.

## Introduction

In 2014, a newly designed case-based seminar was successfully implemented in the subjects of health systems, health economics and public health care (GGG) [1]. The seminar “The Lonely Patient” is based on the real patient case of Mr. P. and deals with the German health care system from the patient's perspective. In order to create more space for discussion and exchange among students, the seminar was redesigned on the basis of the Inverted Classroom Method (ICM) [2]. In contrast to the classical lecture, the ICM reverses the order of the self-study phase and the classroom phase. Students acquire factual knowledge on the topic online using various learning materials, e.g. video podcasts, professional articles, etc., at their own pace and independent of time and location. According to some studies, ICM has so far shown good results in terms of student motivation, commitment and learning outcomes [2], [3], [4], [5], [6], [7]. ICM has also been used in medical education [8], [9], [10], [11], [12].

In the winter semester of 2018/19, a 15-minute, ICM-based online self-learning phase was developed for the seminar “The Lonely Patient”. During this phase, students are first given an overview of the patient's history and are expected to acquire factual knowledge independently by means of defined learning goals. For this purpose, the existing written teaching and learning materials (patient case, factsheets) were transformed into two to three minute instructional videos. In the face-to-face session, the topics of the videos are discussed in greater depth with the help of flipchart summaries, keynote speeches by the lecturers, group discussions, and a quiz. In accordance with the ICAP model (Interactive, Constructive, Active, Passive), this should lead to increased interaction between students and lecturers [13]. According to the ICAP model, active learning leads to a more sustainable acquisition of knowledge, it promotes the ability to solve problems and has a positive effect on students’ motivation for learning. Furthermore, activities and interactions create a learner-centered environment and are based on constructivist teaching rather than a direct, mainly one-sided, lecturer-centered approach to teaching [14]. Quizzes were used as an additional interactive element to further enhance student interaction. 

## Project description

Due to the COVID-19 pandemic, new, purely digital teaching formats had to be developed quickly in the sense of emergency remote teaching [15]. The relatively high additional expenditure for digital teaching could be reduced by the already existing ICM within the framework of the seminar “The Lonely Patient”. The online self-learning phase could be reused directly. The adjustments therefore related to the previous traditional classroom-based course part, which had to be adapted. In order to keep the interaction in pure online teaching as high as possible, the course concept was adapted according to the specifications of the online Inverted Classroom Models [16]. Synchronous teaching was conducted with the video conference system ZOOM. The gamified Audience Response System (ARS) Kahoot! was used to activate students. This is an online based quiz system with points, badges and leaderboards. The positive effect of the use of gamification has already been proven in several studies [16], [17]. The use of ARS and especially the use of Kahoot! could also be proven in some studies as conducive to learning [18]. During the synchronous event, students were repeatedly called upon to answer questions about the event and the originally used quiz. 

## Methodology

The questionnaire was developed on the basis of the ICAP model and comprises 28 evaluation questions that, among other things, record the students’ own learning process. The questionnaire was used at the end of the course via QR code or link (n=202; response rate: 41%).

## Results

Based on the initial evaluation results and feedback rounds with students, it can be cautiously concluded that the use of the online Inverted Classroom Model does not lead to worse results in subjectively perceived learning success and interactivity during classroom instruction compared to previous traditional classroom-based semesters. Thus, in the overall assessment, the seminar is rated slightly better on average than the previous semester. Brief rounds of feedback directly following the seminar confirmed this result (“What did you particularly like about the seminar?”): Students gave positive feedback regarding the course concept and especially emphasized the use of Kahoot! Students also indicated that they preferred the synchronous course format over asynchronous digital teaching. It should also be noted that the response rate of the online evaluation was higher compared to the traditional classroom-based courses of previous semesters or compared to asynchronous teaching.

## Discussion

By using the online Inverted Classroom Model and by means of the planned interactive components (Kahoot!), the interaction with the student could be kept at the same high level as in a classroom format of the same course. The disadvantages of pure online teaching without activating elements could thus be reduced. 

## Conclusion

By using the online Inverted Classroom method, digital teaching could be continued successfully during the COVID-19 summer semester. It also seems that the disadvantages of the pure online teaching, like the reduced or missing interaction with the students could be compensated. According to the ICAP model, the learning success of students could be guaranteed or at least not worsened to a large extent. In the winter semester 2020/21, this course will probably be held in digital form again. It is planned to implement the course concept with an ARS that contains reflective elements compared to Kahoot! Further evaluation will show whether these first positive results can be confirmed. The present concept can theoretically be used in a slightly modified version in the future. 

## Competing interests

The authors declare that they have no competing interests. 
